# Supplementary material for: Antimicrobial Activity of Dihydroisocoumarin Isolated from Wadi Lajab Sediment-Derived Fungus Penicillium chrysogenum: In Vitro and In Silico Study
Source: Molecules. 2022 Jun 6;27(11):3630. doi: 10.3390/molecules27113630 (PMC9182410; doi:10.3390/molecules27113630)
Supplement: Supplementary file 1 [file molecules-27-03630-s001.zip › molecules-1682360-supplementary.pdf]

Supplementary material

Table S1: Molecular docking parameters for the interaction of ligands with NDM-1

Donor atom	Acceptor atom	Distance (Å)	Type of interaction	Docking energy, Δ*G* (kcal mol^{-1})	Binding affinity, K_d (M^{-1})
Meropenem (Control)					
Zn<br>Gln123:HN<br>Asn220:HD21<br>His189:CE1<br>His250	Asp124:OD1<br>Lig:O<br>Lig:O<br>Lig:O<br>Lig:C	2.65268<br>1.83673<br>1.74830<br>3.59611<br>4.45873	Electrostatic<br>Hydrogen Bond<br>Hydrogen Bond<br>Hydrogen Bond<br>Hydrophobic (Pi-Alkyl)	-7.9	6.2×10^5
Compound 1					
Gln123:HN<br>Lig:H<br>Trp93<br>Lig<br>Lig:C<br>Phe70<br>Trp93	Lig:O<br>Asp124:OD2<br>Lig<br>Val73<br>Val73<br>Lig:C<br>Lig	2.23140<br>2.67619<br>4.84540<br>4.90669<br>4.16146<br>5.23287<br>5.22239	Hydrogen Bond<br>Hydrogen Bond<br>Hydrophobic (Pi-Pi T-<br>shaped)<br>Hydrophobic (Alkyl)<br>Hydrophobic (Alkyl)<br>Hydrophobic (Pi-Alkyl)<br>Hydrophobic (Pi-Alkyl)	-5.7	1.5×10^4
Compound 2					
Lig:H<br>Lig:H<br>Asp124:OD2<br>Lig:C	His120:NE2<br>Asp124:OD1<br>Lig<br>Val73	2.68764<br>2.55858<br>3.86467<br>4.40664	Hydrogen Bond<br>Hydrogen Bond<br>Electrostatic (Pi-Anion)<br>Hydrophobic (Alkyl)	-6.0	2.5×10^4
Compound 3					
Lig:H<br>Lig:H<br>Lig:H<br>Asp124:OD2<br>Lig:H<br>Lig:C<br>Trp93<br>Trp93<br>Lig	His250:NE2<br>His120:NE2<br>His189:NE2<br>Lig<br>His250<br>Leu65<br>Lig<br>Lig:C<br>Cys208	2.40288<br>2.81175<br>2.30996<br>3.79230<br>2.79062<br>5.15741<br>4.84339<br>5.18681<br>5.48747	Hydrogen Bond<br>Hydrogen Bond<br>Hydrogen Bond<br>Electrostatic (Pi-Anion)<br>Hydrogen Bond (Pi-<br>Donor)<br>Hydrophobic (Alkyl)<br>Hydrophobic (Pi-Alkyl)<br>Hydrophobic (Pi-Alkyl)<br>Hydrophobic (Pi-Alkyl)	-6.0	2.5×10^4
Compound 4					
Lig:H<br>Lig:H	His120:NE2<br>Asp124:OD1	2.59981<br>2.60945	Hydrogen Bond<br>Hydrogen Bond	-6.2	3.5×10^4

Asp124:OD2	Lig	3.78924	Electrostatic (Pi-Anion)		
Trp93	Lig	4.89358	Hydrophobic (Pi-Pi T-shaped)		
Lig:C	Leu65	5.18009	Hydrophobic (Alkyl)		
Trp93	Lig:C	5.14392	Hydrophobic (Pi-Alkyl)		
Lig	Cys208	5.49731	Hydrophobic (Pi-Alkyl)		
Compound 5					
Lig:H	His250:NE2	2.37525	Hydrogen Bond	-6.2	3.5 × 10^4
Lig:H	His120:NE2	2.85780	Hydrogen Bond		
Lig:H	His189:NE2	2.77597	Hydrogen Bond		
His189:CE1	Lig:O	3.56765	Carbon Hydrogen Bond		
Asp124:OD2	Lig	3.88985	Electrostatic (Pi-Anion)		
Lig:Cl N	His250	3.79463	Hydrophobic (Pi-Sigma)		
Lig:C	Val73	4.27020	Hydrophobic (Alkyl)		
Lig	Val73	5.37177	Hydrophobic (Pi-Alkyl)		

Table S2: Molecular docking parameters for the interaction of ligands with CTX-M-15.

Donor atom	Acceptor atom	Distance (Å)	Type of interaction	Docking energy, ΔG (kcal mol^{-1})	Binding affinity, K_d (M^{-1})
Avibactam (control)					
Asn104:HD22	Lig:O	2.61748	Hydrogen Bond	-6.8	9.7 × 10^4
Lys234:HZ1	Lig:O	2.51108	Hydrogen Bond		
Lig:H	Thr216:O	2.78293	Hydrogen Bond		
Compound 1					
Ser70:HG	Lig:O	2.95585	Hydrogen Bond	-6.7	8.2 × 10^4
Asn132:HD22	Lig:O	1.86264	Hydrogen Bond		
Asn170:HD22	Lig:O	2.86371	Hydrogen Bond		
Compound 2					
Ser70:HG	Lig:O	2.85091	Hydrogen Bond	-6.5	5.8 × 10^4
Ser130:HG	Lig:O	2.38463	Hydrogen Bond		
Asn132:HD22	Lig:O	1.86294	Hydrogen Bond		
Lig:H	Asn132:OD1	2.13260	Hydrogen Bond		
Lig:H	Glu166:OE2	3.06227	Hydrogen Bond		
Tyr105	Lig	4.10881	Hydrophobic (Pi-Alkyl)		
Tyr105	Lig:C	4.28344	Hydrophobic (Pi-Alkyl)		
Compound 3					
Ser70:HG	Lig:O	2.91922	Hydrogen Bond	-6.5	5.8 × 10^4
	Lig:O	1.89778	Hydrogen Bond		

Asn132:HD22<br>Asn170:HD22<br>Lig:H<br>Lig:H	Lig:O<br>Ser130:O<br>Asn132:OD1	2.85566<br>2.39515<br>2.63829	Hydrogen Bond<br>Hydrogen Bond<br>Hydrogen Bond		
Compound 4					
Lys73:HZ3<br>Ser130:HG<br>Asn132:HD22<br>Asn170:HD22<br>Lig:H	Lig:O<br>Lig:O<br>Lig:O<br>Lig:O<br>Asn132:OD1	2.95838<br>2.25025<br>1.89371<br>2.85984<br>2.31038	Hydrogen Bond<br>Hydrogen Bond<br>Hydrogen Bond<br>Hydrogen Bond<br>Hydrogen Bond	-6.8	9.7×10^4
Compound 5					
Ser130:HG<br>Asn132:HD22<br>Asn170:HD22<br>Lig:H<br>Lig:H	Lig:O<br>Lig:O<br>Lig:O<br>Ser130:O<br>Asn132:OD1	2.26789<br>1.82750<br>2.85047<br>2.33443<br>2.65272	Hydrogen Bond<br>Hydrogen Bond<br>Hydrogen Bond<br>Hydrogen Bond<br>Hydrogen Bond	-6.7	8.2×10^4

Table S3: Molecular docking parameters for the interaction of ligands with OXA-48

Donor atom	Acceptor atom	Distance (Å)	Type of interaction	Docking energy, ΔG (kcal mol^{-1})	Binding affinity, K_d (M^{-1})
			Imipenem (control)		
Arg250:HH22	Lig:O	1.774	Hydrogen Bond	-6.1	2.9 × 10^4
Lig:H	Ser70:OG	1.827	Hydrogen Bond		
Lig:H	Thr209:OG1	2.263	Hydrogen Bond		
Lig:HN	Lys116:O	2.315	Hydrogen Bond		
Lig:H	Thr209:OG1	2.377	Hydrogen Bond		
Lig:H	Tyr211:O	2.325	Hydrogen Bond		
			Compound 1		
Tyr211:HN	Lig:O	2.447	Hydrogen Bond	-6.3	4.1 × 10^4
Val120:CG2	Lig	3.779	Hydrophobic (Pi-Sigma)		
Lig:C	Ile102	5.133	Hydrophobic (Alkyl)		
Tyr211	Lig:C	5.279	Hydrophobic (Pi-Alkyl)		
			Compound 2		
Trp105:HE1	Lig:O	2.316	Hydrogen Bond	-6.3	4.1 × 10^4
Arg250:HH12	Lig:O	2.999	Hydrogen Bond		
Arg250:HH22	Lig:O	1.750	Hydrogen Bond		
			Compound 3		
Tyr211:HN	Lig:O	2.513	Hydrogen Bond	-6.2	3.5 × 10^4
Lig:H	Tyr211:O	2.955	Hydrogen Bond		
Val120:CG2	Lig	3.845	Hydrophobic (Pi-Sigma)		
Lig:C	Ile102	5.058	Hydrophobic (Alkyl)		
Tyr211	Lig:C	5.272	Hydrophobic (Pi-Alkyl)		
Lig	Leu158	5.443	Hydrophobic (Pi-Alkyl)		
			Compound 4		
Tyr211:HN	Lig:O	2.909	Hydrogen Bond	-6.4	4.9 × 10^4
Arg250:HH12	Lig:O	1.885	Hydrogen Bond		
Lig:H	Thr209:OG1	2.300	Hydrogen Bond		
Tyr211	Lig	5.802	Hydrophobic (Pi-Pi T-shaped)		
Tyr211	Lig	5.532	Hydrophobic (Pi-Pi T-shaped)		
Tyr211	Lig:Cl	5.205	Hydrophobic (Pi-Alkyl)		
Lig	Leu247	5.026	Hydrophobic (Pi-Alkyl)		
			Compound 5		

Tyr211:HN<br>Arg214:HE<br>Tyr211<br>Lig:C<br>Lig:Cl<br>Tyr211<br>Lig<br>Lig	Lig:O<br>Lig:O<br>Lig<br>Ile102<br>Ile102<br>Lig:C<br>Val120<br>Leu158	2.614<br>2.339<br>5.366<br>5.150<br>5.063<br>5.077<br>4.999<br>5.247	Hydrogen Bond<br>Hydrogen Bond<br>Hydrophobic (Pi-Pi T-shaped)<br>Hydrophobic (Alkyl)<br>Hydrophobic (Alkyl)<br>Hydrophobic (Pi-Alkyl)<br>Hydrophobic (Pi-Alkyl)<br>Hydrophobic (Pi-Alkyl)	-6.2	3.5×10^4
